# Unfertilized and Washed Eri Silkworm Eggs as Superior Hosts for Mass Production of *Trichogramma* Parasitoids

**DOI:** 10.3390/insects16080751

**Published:** 2025-07-22

**Authors:** Yue-Hua Zhang, Ji-Zhi Xue, He-Ying Qian, Qing-Rong Bai, Tian-Hao Li, Jian-Fei Mei, Lucie S. Monticelli, W. M. W. W. Kandegama, Lian-Sheng Zang

**Affiliations:** 1 Jiangsu Key Laboratory of Sericultural Biology and Biotechnology, School of Biotechnology, Jiangsu University of Science and Technology, Zhenjiang 212100, China; 2 State Key Laboratory of Green Pesticides, Guizhou University, Guiyang 550025, China; 3 Guizhou Zhuohao Agricultural Technology Co., Ltd., Zunyi 563000, China; 4 INRAE, CNRS, UMR-ISA, Université Côte d’Azur, 06000 Nice, France; 5 Department of Horticulture and Landscape Gardening, Faculty of Agriculture and Plantation Management, Wayamba University of Sri Lanka, Makandura, Gonawila 60170, Sri Lanka

**Keywords:** parasitoid, factitious host, biological control, host treatment, host suitability, mass production

## Abstract

*Trichogramma* wasps are highly effective biological control agents, offering an environmentally sustainable solution for pest management through their parasitism of insect eggs. This study evaluates the parasitism performance of six *Trichogramma* species—*T. dendrolimi*, *T. chilonis*, *T. leucaniae*, *T. ostriniae*, *T. japonicum*, and *T. pretiosum*—on five treatments of Eri silkworm (ES) eggs, a potential alternative to the large eggs of *Antheraea pernyi* for mass rearing. The ES egg treatments included the following: manually extracted, unfertilized, and washed eggs (MUW); naturally laid, unfertilized, and washed eggs (NUW); naturally laid, unfertilized, and unwashed eggs (NUUW); naturally laid, fertilized, and washed eggs (NFW); and naturally laid, fertilized, and unwashed eggs (NFUW). The results demonstrate that all *Trichogramma* species, except *T. japonicum*, successfully parasitized ES eggs across all treatments. Notably, washed eggs consistently supported higher parasitism and emergence rates compared to unwashed eggs, while unfertilized eggs outperformed fertilized eggs in these metrics. Among the treatments, unfertilized and washed eggs (MUW and NUW) exhibited the shortest pre-emergence time and the highest number of emerged adults, with no significant differences in female progeny ratios across most species. A striking exception was *T. dendrolimi*, which showed a significantly higher female offspring ratio in the MUW treatment. These findings highlight that MUW eggs of ES are a highly suitable alternative host for the mass production of *Trichogramma* wasps. This study provides critical insights for optimizing host egg treatments to enhance the efficiency of *Trichogramma*-based biological control programs.

## Simple Summary

The large eggs of Chinese oak silkworm (*Antheraea pernyi*) have long been employed in China as an effective factitious host for mass-rearing *Trichogramma* parasitoids in agricultural and forestry pest management. Recent attention has turned to the Eri silkworm (*Samia ricini*) as a promising alternative host, owing to it possessing similarly large eggs with a broader geographical distribution. This study systematically assesses the suitability of six dominant *Trichogramma* species reared under five distinct treatments of *S. ricini* eggs. We identify the optimal egg treatment protocol to enhance *Trichogramma* productivity, thereby providing a scalable solution for industrial-scale parasitoid rearing. Our findings contribute to the optimization of sustainable biological control strategies against lepidopteran pests in agricultural and forestry ecosystems.

## 1. Introduction

Biological control is a sustainable pest management strategy that leverages natural food chain dynamics and ecological interactions to suppress target pest populations [1]. This approach helps to minimize the use of chemical pesticides that are responsible for environmental pollution and side effects in non-target organisms and ecosystem services, and it helps to sustain biodiversity, making it an environmentally friendly alternative for pest control [2,3]. *Trichogramma* (Hymenoptera: Trichogrammatidae), a genus of egg parasitoids, are known to parasitize eggs of multiple different agricultural pests [4,5,6,7,8,9]. As highly effective natural enemies of agricultural and forestry pests, *Trichogramma* wasps play a pivotal role in integrated pest management [10]. Their deployment in biological control programs not only reduces reliance on chemical pesticides—ensuring pollution-free and residue-free green pest control—but also offers sustainable, long-term suppression of pest populations. This aligns with the growing global demand for safe, healthy, and eco-friendly agricultural products [11,12].

Globally, *Trichogramma* is the most extensively utilized parasitoid wasp for controlling lepidopteran pests and is amenable to large-scale factory production [13,14]. The alternative hosts employed for the mass rearing of *Trichogramma* are termed factitious hosts, which can be classified into two categories based on size: large eggs (e.g., Chinese oak silkworm (*Antheraea pernyi* Guérin-Méneville) and Eri silkworm (*Samia ricini* William Jones) eggs, which support the development of three or more wasps per egg; and small eggs (e.g., rice moth (*Corcyra cephalonica* Stainton) and angoumois grain moth (*Sitotroga cerealella* Olivier) eggs), which typically yield only one or two wasps per egg [15,16,17]. Ideal factitious hosts should exhibit cost-effectiveness, operational efficiency, high quality, and extended storage viability. However, current rearing technologies relying on *A. pernyi* (large eggs), as well as small eggs including *S. cerealella* and *C. cephalonica*, face notable limitations. *Antheraea pernyi* eggs are constrained by seasonal availability, geographical restrictions, and limited suitability for certain *Trichogramma* species, while small eggs entail high production costs, low yields, and labor-intensive processes [18,19]. Other methodologies, e.g., artificial host eggs, are not practicable yet [20,21,22]. These challenges pose significant barriers to the widespread adoption and scalability of *Trichogramma*-based biological control programs.

The Eri silkworm (*Samia ricini*) (ES) is among the most widely reared silk-producing insects globally, alongside key species such as the domestic silkworm (*Bombyx mori*), *Antheraea pernyi* [23,24], and other regionally high-potential species like *Caligula japonica* [25]. ES offers significant advantages for mass rearing, including low production costs, simple feeding requirements, rapid reproduction, high fecundity, and strong disease resistance, with an annual capacity of 6–7 generations in southern China [26,27]. As a factitious host for *Trichogramma* wasps, ES eggs demonstrate exceptional suitability, yielding 20–30 adult wasps per egg. Field trials have shown outstanding efficacy in sugarcane stem borer control, achieving parasitism rates of 65.65–94.18% [26]. Notably, *Trichogramma chilonis* exhibits significantly higher parasitism rates on ES eggs compared to *A. pernyi* eggs, confirming ES to be a superior alternative host for *Trichogramma* mass production in biological control programs [28].

Host egg characteristics are critical determinants of parasitoid reproductive efficiency, making the selection of optimal factitious hosts a fundamental consideration for successful biological control [29,30]. Several studies have found different suitability of parasitoid wasps for hosts. The characteristics of the host, including egg size, eggshell thickness, nutrient content of the host egg fluid, and age of the eggs, have been demonstrated to influence parasitism by *Trichogramma* [31,32].

*Trichogramma* species demonstrate distinct host egg preferences that are influenced by the nutritional composition and physiological state of the eggs [33]. Notably, studies have shown that *T. dendrolimi* preferentially parasitizes dissected, unfertilized *A. pernyi* eggs, making this combination particularly suitable for mass rearing programs [15]. Previous studies have shown that the host preference of *Trichogramma* can be influenced by the fertilization status of the host eggs. The study revealed that, in no-choice tests, *T. dendrolimi* parasitized significantly more unfertilized eggs than fertilized ones, whereas *T. ostriniae* parasitized significantly more fertilized eggs. In contrast, in choice tests, *T. dendrolimi* preferred unfertilized eggs, while *T. ostriniae* showed no clear preference [34]. Similar preferences have been observed in other egg parasitoids, including *Anastatus gansuensis* sp. Chen & Zang, *Mesocomys trabalae* Yao, Yang and Zhao, *Anastatus fulloi* Sheng & Wang, and *Anastatus meilingensis* Sheng, which exhibit greater host acceptance for unwashed, unfertilized *A. pernyi* eggs [35]. These findings suggest that the differential parasitism performance of *Trichogramma* species may be partly explained by their oviposition preferences, which are influenced by egg fertilization status. Furthermore, the results underscore the importance of host egg pre-treatment in mass rearing and field releases to improve biological control effectiveness.

The large host egg pretreatment methods significantly impact both parasitoid acceptance and offspring performance. Key treatment variables include the method of egg collection (dissected vs. naturally laid), fertilization status, and washing procedure [36]. Currently, *A. pernyi* eggs serve as the primary large-egg intermediate host in commercial production, typically obtained through the dissection of unfertilized female moths followed by water washing [15,35]. However, despite the growing interest in ES eggs as an alternative host, no comprehensive studies have evaluated how different ES egg treatments affect parasitism preferences or offspring performance in *Trichogramma* wasps.

The aim of the present study is to assess the adaptation of six dominant *Trichogramma* wasps, which are widely used in the field, to different treatments of ES eggs, in order to determine the optimal treatment of ES eggs for rearing *Trichogramma*.

## 2. Materials and Methods

### 2.1. Hosts

An Eri silkworm (ES, *Samia ricini*) colony was maintained at our research facility in Guiyang City, Guizhou Province, China. Larvae were reared under controlled laboratory conditions using fifth instar. Following cocoon formation, pupae were transferred to a moth emergence chamber maintained at 25 ± 3 °C and 80 ± 5% relative humidity under natural photoperiod conditions. Daily monitoring commenced 10–11 days post-cocooning to record adult emergence. Randomly selected newly emerged (<8 h) moths were used as experimental samples. We established five distinct ES egg treatment protocols: (1) Manually extracted unfertilized washed eggs (MUW): Eggs were dissected from unmated female abdomens, then washed and dried (washed with distilled water and then air-dried under the above controlled conditions). (2) Naturally laid unfertilized unwashed eggs (NUUW): Collected after 24 h oviposition by unmated females. (3) Naturally laid unfertilized washed eggs (NUW): NUUW eggs that were subsequently washed. (4) Naturally laid fertilized unwashed eggs (NFUW): Collected after 24 h oviposition from mated females. (5) Naturally laid fertilized washed eggs (NFW): NFUW eggs that were subsequently washed.

### 2.2. Parasitoids

Six *Trichogramma* species were obtained for this study: *T. chilonis*, *T. dendrolimi*, and *T. japonicum* were field-collected from *Chilo suppressalis* Walker egg masses in Changchun City, Jilin Province, China (43.89° N, 125.32° E); *T. leucaniae* and *T. ostriniae* were collected from *Leguminivora glycinivorella* Matsumura eggs in Heihe City, Heilongjiang Province, China (50.22° N, 127.53° E); and *T. pretiosum* was obtained from Nanjing Agricultural University, China, as a laboratory strain. All field-collected species were authenticated using morphological examination of male genital capsules and rDNA-ITS2 sequence analysis (GenBank accession numbers: *T. chilonis*: FR750277; *T. dendrolimi*: FR750279; *T. leucaniae*: HG518480; *T. ostriniae*: HE648326; *T. japonicum*: FN822757). All *Trichogramma* species were reared for up to 10 generations on *C. cephalonica* eggs. Rearing was conducted under controlled environmental conditions: temperature: 26 ± 1 °C; relative humidity: 75 ± 5%; photoperiod: 14L:10D (light–dark).

### 2.3. Parasitic Suitability of Differently Processed ES Eggs for Trichogramma Wasps

A non-toxic adhesive was used to attach five ES eggs to strips of paper card with 1 cm distance between the eggs, and then one egg card was transferred to glass tubes of 3.5 cm in diameter and 10 cm in length, and fifteen newly emerged *Trichogramma* adult females were introduced, all of which mated within 8 h of their emergence. After 24 h, the parasitoid wasps were removed. The parasitized eggs were placed in an incubator (MLR-351H, SANYO Electric Co., Ltd., Osaka, Japan) at a temperature of 25 ± 1 °C, relative humidity of 70 ± 5%, and light of 14L:10D to continue their development. After 6 days, the parasitism of the ES eggs of the different treatments was observed and recorded (gray egg were the parasitized eggs), and the parasitism rate was counted. After wasps had emerged, the emergence rate, female rate (number of females/total number of emerged wasps), number of emerged adult wasps/egg, and pre-emergence time (time elapsed from the start of parasitism to the emergence of the adult wasp) of *Trichogramma* wasps in each treatment group were recorded every day, and each treatment group was replicated 30 times.

### 2.4. Data Analysis

Linear models (LMs) were used to analyze the pre-emergence time and the number of emerged adults per egg, as residuals for these variables met the assumptions of normality. For parasitism rate, emergence rate, and female ratio, which are proportions, generalized linear models (GLMs) with a binomial distribution were applied. The explanatory variables included parasitoid species (PS), extraction (E), fertilization (F), washing (W), and their interactions (PS × E, PS × F, PS × W, PS × F × W).

Model significance was assessed using analysis of variance (ANOVA or deviance analysis, depending on the model type). Because several interactions were statistically significant, multiple comparisons were conducted using Tukey’s HSD test on the full dataset with the ‘multcomp’ package in R (version 4.1.1). All figures were produced using the ‘ggplot2’ package.

## 3. Results

### 3.1. Effect of Different ES Egg Treatments on Trichogramma Parasitism Rates

**Figure 1:**
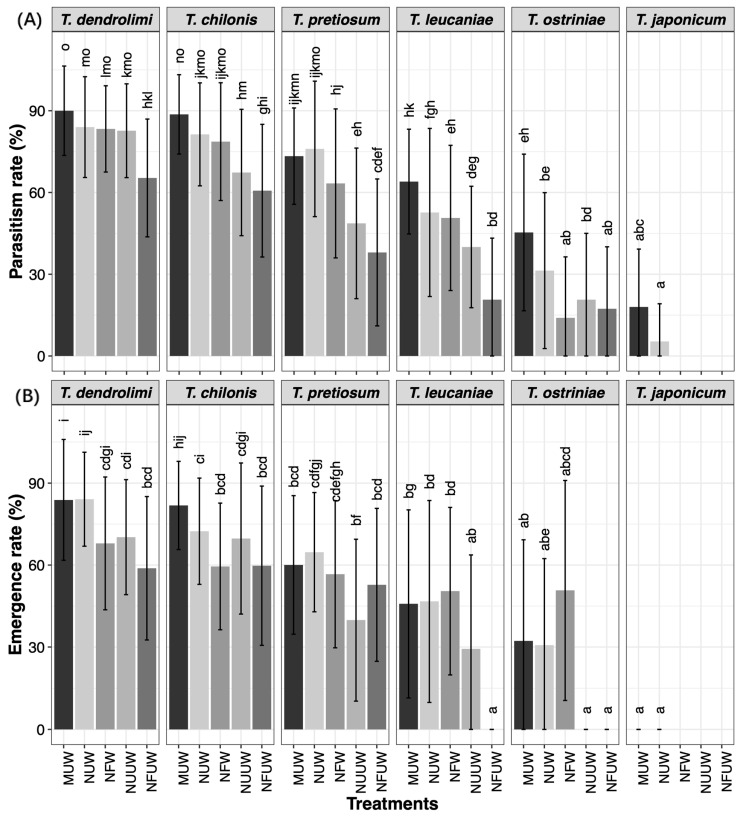
Mean (±SE) of parasitism rate (**A**) and emergence rate (**B**) of *Trichogramma* of ES eggs in different treatments. Different letters on the bars indicate significant differences (*p* < 0.05, Tukey’s HSD test).

The parasitism rate was significantly influenced by the parasitoid species (PS), host egg treatments (E, F, W), and their interactions (Figure 1, Table 1). Notably, significant interactions were observed between PS and W, as well as between PS, F, and W.

Parasitism variation by wasp species: *T. dendrolimi* parasitized MUW, NUW, and NFW eggs at significantly higher rates than NUUW and NFUW eggs. *T. chilonis*, *T. leucaniae*, *T. ostriniae*, and *T. japonicum* exhibited the highest parasitism rates in MUW eggs compared to other treatments. *T. pretiosum* showed significantly higher parasitism in MUW and NUW eggs than on other treatments.

Host egg-specific parasitism patterns: MUW eggs: *T. dendrolimi* (89.3%) and *T. chilonis* (86.7%) achieved the highest parasitism rates, while *T. japonicum* had the lowest. NUW eggs: The highest parasitism was observed for *T. dendrolimi* (84.0%), followed by *T. chilonis* (81.3%) and *T. pretiosum* (76.0%). *T. leucaniae*, *T. ostriniae*, and *T. japonicum* exhibited progressively lower rates. NFW eggs: *T. dendrolimi* (88%) and *T. chilonis* (78.6%) outperformed *T. pretiosum* (63.3%), *T. leucaniae* (50.7%), and *T. ostriniae* (12.7%). Notably, *T. japonicum* failed to parasitize NFW eggs (0%). NUUW eggs: *T. dendrolimi* and *T. chilonis* showed the highest parasitism (77.3%), significantly surpassing other species. *T. japonicum* again showed no parasitism. NFUW eggs: *T. dendrolimi* (63.3%) and *T. chilonis* (60.7%) led to parasitism, followed by *T. pretiosum*, *T. leucaniae*, and *T. ostriniae*.

### 3.2. Parasitic Suitability of Differently Treated ES Eggs for Trichogramma Wasps

The parasitic adaptation of *Trichogramma* wasps to various ES egg treatments was significantly affected by host egg treatment, wasp species, and their interactions (Table 1 and Table 2). The key parameters of parasitism success—emergence rate, number of emerged adults, female ratio, and pre-emergence time—showed distinct patterns across treatments and species.

Suitability variation by wasp species: *T. dendrolimi* demonstrated optimal emergence rate on NUW eggs (84.2%; *p* < 0.05), and produced maximum adult yield in NUW treatment (30.7 adults; *p* = 0.021), highest female ratio from MUW eggs (88.7%; *p* = 0.048), and shortest pre-emergence time on MUW, NUW, and NFW eggs compared to NFUW (*p* < 0.05). *T. chilonis* achieved peak emergence rate on MUW eggs (81.8%; *p* < 0.001), maximum adult production in NUW treatment (29.9 adults; *p* < 0.05), consistently high female ratios (>80%) across all treatments (*p* = 0.08), and a stable pre-emergence time (10.75–11.0 days; *p* = 0.249). *T. pretiosum* demonstrated highest emergence rate on NUW eggs (64.7%; *p* < 0.05), maximum adult production in MUW (23.8) and NUW (24.4) treatments (*p* < 0.05), and significantly shorter pre-emergence time on MUW, NUW, NFW, and NUUW vs. NFW (*p* < 0.05) eggs. *T. leucaniae* failed to emerge from NFUW eggs (0% emergence), and demonstrated higher emergence rates on MUW, NUW, and NFW vs. NUUW (*p* < 0.05) eggs, consistent adult production (23.7–25.3) (*p* = 0.506) and female ratios (83.7–87.8%) (*p* = 0.191) across treatments, and shorter pre-emergence on MUW/NUW (14.3 days) vs. NFW/NUUW (*p* < 0.05) eggs. *T. ostriniae* demonstrated similar emergence rates across MUW, NUW, and NFW (*p* = 0.481) eggs, highest adult production in MUW (19.5) and NFW (16.9) (*p* < 0.05) eggs, significantly higher female ratio in MUW (79.6%) vs. NUW/NFW (*p* < 0.05) eggs, and shorter pre-emergence in MUW (14.4 days) vs. NUW (15.2) and NFW (15.3) (*p* < 0.05) eggs.

Host egg-specific suitability patterns: MUW eggs: *T. dendrolimi* and *T. chilonis* exhibited the highest emergence rates, followed by *T. pretiosum* and *T. leucaniae*, with the lowest rates observed in *T. ostriniae* (*p* < 0.05). *T. chilonis* produced significantly more adult wasps than other species (*p* < 0.05). *T. pretiosum* showed complete female production (thelytoky) (*p* < 0.05). *T. dendrolimi* and *T. chilonis* had shortest pre-emergence periods (*p* < 0.05). NUW eggs: *T. dendrolimi* exhibited a significantly higher emergence rate compared to other species (*p* < 0.05). *T. dendrolimi* and *T. chilonis* showed highest adult production (*p* < 0.05). The highest female ratio among *T. pretiosum* had highest female ratio, followed by *T. chilonis*, *T. dendrolimi*, and *T. leucaniae*, with the lowest in *T. ostriniae* (*p* < 0.001). *T. dendrolimi* and *T. chilonis* developed fastest, while *T. ostriniae* was slowest (*p* < 0.05). NFW eggs: *T. dendrolimi* exhibited the highest emergence rate (*p* < 0.05) and produced the most adults (*p* < 0.05). *T. pretiosum* had the highest female ratio, while *T. ostriniae* had the lowest (*p* < 0.05). *T. dendrolimi* and *T. chilonis* developed significantly faster than others (*p* < 0.05). NUUW eggs: *T. dendrolimi* and *T. ostriniae* had significantly higher emergence rates compared to *T. pretiosum* and *T. leucaniae*, while *T. ostriniae* failed to emerge (*p* < 0.05). *T. dendrolimi* and *T. ostriniae* also produced more adults than *T. leucaniae*, while *T. pretiosum* produced the fewest (*p* < 0.05). *T. pretiosum* showed the highest female ratio (*p* < 0.001). *T. dendrolimi* and *T. ostriniae* developed fastest (*p* < 0.05). NFUW eggs: There were no significant differences in emergence rates among *T. dendrolimi*, *T. chilonis*, and *T. pretiosum,* while *T. leucaniae* and *T. ostriniae* failed to emerge (*p* = 0.059). *T. dendrolimi* and *T. ostriniae* produced more adults than *T. pretiosum* (*p* < 0.05). *T. pretiosum*, *T. dendrolimi*, and *T. ostriniae* had the highest female proportion (*p* < 0.001). *T. dendrolimi* and *T. chilonis* developed significantly faster than *T. pretiosum* (*p* < 0.05).

## 4. Discussion

The parasitism efficiency of *Trichogramma* wasps is governed by both extrinsic (e.g., humidity, light, temperature) and intrinsic host factors (e.g., egg age, size, surface chemistry, and nutrient content) [37]. For mass rearing on factitious hosts, host egg acceptability and suitability are critical determinants of success [38,39]. Our study reveals that all tested *Trichogramma* species—except *T. japonicum*—successfully parasitized all egg treatments, with *T. japonicum* restricted to unfertilized washed eggs and failing to produce viable offspring. Notably, all species exhibited a pronounced preference for manually extracted, unfertilized washed eggs, aligning with prior reports that host fertilization status profoundly impacts parasitism success and offspring fitness [40,41]. While female ratios and pre-emergence times were similar between fertilized and unfertilized eggs, emergence rates were consistently higher in unfertilized eggs.

Insect egg surface secretions perform three critical biological functions: (1) lubrication during oviposition through the reproductive tract, (2) substrate adhesion for the egg’s attachment to plant surfaces, and (3) protective barrier formation against abiotic stressors (desiccation, precipitation) and biotic threats (microbial pathogens, predators) [42,43]. The surface distribution of these secretions governs their protective efficacy, influencing the wasp’s oviposition capability and, ultimately, parasitism outcomes [44]. This aligns with previous findings of light-yellow secretions coating ES eggs [45]. Our results demonstrate significantly higher parasitism and emergence rates in *Trichogramma* wasps on washed eggs versus unwashed eggs. This enhanced performance likely results from the elimination of surface secretions during washing, which presumably reduces the physical and chemical barriers to oviposition. Furthermore, consistent with previous reports [46,47], we confirm that host egg characteristics—particularly shell hardness and thickness—constitute critical limiting factors for parasitoid success, with thicker or harder eggshells substantially impeding parasitism efficiency and compromising mass rearing outcomes. Notably, in washed ES eggs, *Trichogramma* exhibited significantly greater parasitism rates in manually extracted eggs compared to naturally laid eggs. This preference may reflect (1) the reduced shell thickness in processed eggs, facilitating ovipositor penetration, and (2) the partial removal of outer shell layers during the extraction procedure. Together, these modifications appear to create more favorable conditions for parasitoid development.

The nutritional quality of host eggs fundamentally determines parasitoid wasp development success, serving as the exclusive nutrient source for larval growth [48]. During embryogenesis, fertilized eggs undergo dynamic biochemical changes—including depletion of sugars, polyols, and proteins—that alter their nutritional profile and consequently impact parasitoid development [49]. Our findings reveal that *T. dendrolimi* preferentially parasitizes unfertilized eggs, contrasting with reports of other *Trichogramma* species favoring fertilized dinoflagellate eggs [50]. This nutritional paradox warrants further investigation into the physiological and molecular differences between fertilized and unfertilized *Equisetum* eggs, particularly regarding why unfertilized eggs appear nutritionally inferior for parasitoid development.

Previous studies document *T. ostriniae*’s inability to develop in *A. pernyi* eggs and *T. leucaniae*’s poor performance on this host [51]. Our results demonstrate that both species show markedly improved adaptability to ES eggs, suggesting that multi-generational domestication could optimize rearing protocols. For *T. japonicum*, the physical constraints of *A. pernyi* eggs (surface structure, shell thickness) completely prevent parasitism. Chinese researchers have overcome similar limitations using multiparasitism technology, where *T. dendrolimi* and *T. chilonis* create oviposition holes, enabling *T. ostriniae* emergence [18]. Although *T. japonicum* can parasitize MUW and NUW ES eggs in our study, emergence failure suggests that similar co-parasitism strategies may be required.

The established that *A. pernyi*-based production infrastructure can be modified for ES eggs by adjusting processes to accommodate their distinct characteristics. Such adaptations would leverage existing systems while improving the cost-efficiency and scalability of biological control programs. Future development of *T. japonicum* products may depend on (1) multiparasitism techniques with compatible *Trichogramma* species and (2) the optimization of ES egg processing methods to enhance parasitoid emergence success.

## Figures and Tables

**Table 1:** Linear models (LMs) and generalized linear models (GLMs) were used to assess the effects of parasitoid wasp species (PS), host egg treatments—extraction (E), fertilization (F), and washing (W)—and their interactions on several biological indexes of *Trichogramma*.

Parameters	Source	Df	F/*χ*2	*p* Value
Parasitism rate (%)	PS	5	889.75	<0.001
	E	1	125.79	<0.001
	F	1	30.61	<0.001
	W	1	86.67	<0.001
	PS × E	5	10.42	0.064
	PS × F	4	2.87	0.580
	PS × W	4	13.28	<0.001
	PS × F × W	4	13.15	0.011
Emergence rate (%)	PS	5	298.368	<0.001
	E	1	17.079	<0.001
	F	1	13.435	<0.001
	W	1	43.137	<0.001
	PS × E	5	9.934	0.077
	PS × F	4	9.425	0.051
	PS × W	4	38.813	<0.001
	PS × F × W	4	19.269	<0.001
No. of emerged adult/egg	PS	4	115.8651	<0.001
	E	1	18.3954	<0.001
	F	1	16.895	<0.001
	W	1	20.6282	<0.001
	PS × E	4	5.268	<0.001
	PS × F	4	5.9848	<0.001
	PS × W	3	4.9547	<0.001
	PS × F × W	2	1.9059	0.150
Female rate (%)	PS	4	928.98	<0.001
	E	1	11.95	<0.001
	F	1	4.05	0.044
	W	1	0.07	0.793
	PS × E	4	9.13	0.058
	PS × F	4	3.1	0.540
	PS × W	3	3.7	0.296
	PS × F × W	2	2.78	0.249
Pre-emergence time (d)	PS	4	1276.7529	<0.001
	E	1	29.4351	<0.001
	F	1	9.0794	0.003
	W	1	29.8817	<0.001
	PS × E	4	7.6765	<0.001
	PS × F	4	1.4216	0.225
	PS × W	3	2.2331	0.0839
	PS × F × W	2	1.6543	0.192

**Table 2:** Effects of ES egg treatments on emergence rate, number of emerged adults, female rate, and pre-emergence time in *Trichogramma* species. Values (mean ± SE) followed by different lower-case letters indicate significant differences among these parasitoid species on the host eggs (*p* < 0.05, Tukey’s HSD test). (*T. dendrolim*: TD; *T. chilonis*: TC; *T. pretiosum*: TP; *T. leucaniae*: TL; *T. ostriniae*: TO).

Parameters	Parasitoid Species	Host Egg Treatments				
		MUW	NUW	NFW	NUUW	NFUW
No. of emerged adult/egg	TD	28.23 ± 0.70 abcde	30.73 ± 1.22 a	29.23 ± 0.92 abc	29.37 ± 0.84 ab	26.5 ± 0.77 abcde
	TC	29.07 ± 1.79 abcd	29.9 ± 1.18 ab	25.6 ± 0.90 bcde	29.4 ± 0.90 ab	25.23 ± 0.92 bcde
	TP	23.77 ± 1.08 efg	24.37 ± 0.96 cdef	19.13 ± 0.88 gh	17.00 ± 0.91 hi	15.60 ± 0.90 hi
	TL	25.3 ± 0.88 bcde	24.97 ± 0.84 bcde	24.27 ± 0.79 def	23.73 ± 0.66 efg	0
	TO	19.53 ± 1.00 fgh	13.37 ± 0.82 i	16.93 ± 0.89 hi	0	0
Female rate (%)	TD	88.49 ± 1.42 e	82.87 ± 1.48 de	85.34 ± 1.64 de	86.21 ± 0.90 de	84.64 ± 1.08 e
	TC	86.69 ± 1.26 e	85.88 ± 0.96 de	82.00 ± 1.00 ade	84.87 ± 1.48 de	83.86 ± 1.65 de
	TP	100.00 ± 0.00 c	100.00 ± 0.00 c	100.00 ± 0.00 c	100.00 ± 0.00 c	100.00 ± 0.00 c
	TL	83.92 ± 1.66 de	87.69 ± 1.19 de	83.93 ± 1.95 de	84.41 ± 1.14 de	--
	TO	79.94 ± 0.91 bd	74.72 ± 1.75 ab	69.30 ± 1.22 b	--	--
Pre-emergence time (d)	TD	10.59 ± 0.15 gh	10.56 ± 0.18 gh	10.28 ± 0.12 h	10.75 ± 0.11 fgh	11.32 ± 0.16 f
	TC	10.89 ± 0.12 fgh	10.75 ± 0.15 fgh	10.76 ± 0.13 fgh	10.92 ± 0.13 fgh	11.01± 0.12 fg
	TP	13.98 ± 0.12 e	14.27 ± 0.12 de	14.41 ± 0.12 bcde	14.33 ± 0.12 bcde	14.92 ± 0.13 abc
	TL	14.33 ± 0.07 bcde	14.29 ± 0.10 cde	14.83 ± 0.12 abcd	14.94 ± 0.10 ab	--
	TO	14.38 ± 0.12 bcde	15.19 ± 0.13 a	15.26 ± 0.09 a	--	--

## Data Availability

The raw data supporting the conclusions of this article will be made available by the authors on request.
